# Neutrophil Elastase Increases Vascular Permeability and Leukocyte Transmigration in Cultured Endothelial Cells and Obese Mice

**DOI:** 10.3390/cells11152288

**Published:** 2022-07-25

**Authors:** Chinchu Jagadan Ushakumari, Qiong L. Zhou, Yu-Hua Wang, Sijia Na, Michael C. Rigor, Cindy Y. Zhou, Max K. Kroll, Benjamin D. Lin, Zhen Y. Jiang

**Affiliations:** 1 Department of Pharmacology & Experimental Therapeutics, School of Medicine, Boston University, Boston, MA 02118, USA; chinchuj@bu.edu (C.J.U.); qzhou@bu.edu (Q.L.Z.); youhua_wang@email.ncu.edu.cn (Y.-H.W.); sijiana@xjtu.edu.cn (S.N.); 2 Whitaker Cardiovascular Institute, School of Medicine, Boston University, Boston, MA 02118, USA; mcr177@rwjms.rutgers.edu (M.C.R.); czhou3@bu.edu (C.Y.Z.); maxkroll@bu.edu (M.K.K.); benlin@bu.edu (B.D.L.)

**Keywords:** neutrophil elastase, vascular endothelial injury, obesity, neutrophils, systemic inflammation

## Abstract

Neutrophil elastase (NE) plays a pivotal role in inflammation. However, the mechanism underlying NE-mediated inflammation in obesity remains unclear. Here, we report that NE activates protease-activated receptor-2 (PAR2), stimulates actin filament (F-actin) formation, decreases intercellular junction molecule VE-cadherin expression, and increases the permeability of human arterial endothelial cells (hECs). NE also prompts degradation of VE-cadherin and its binding proteins p120- and β-catenins via MG132-sensitive proteasomes. NE stimulates phosphorylation of myosin light-chain (MLC) and its regulator myosin phosphatase target subunit-1 (MYPT1), a target of Rho kinase (ROCK). Inhibitors of PAR2 and ROCK prohibit NE-induced F-actin formation, MLC phosphorylation, and VE-cadherin reduction in hECs, and impede monocyte transmigration through hEC monolayer pretreated with either neutrophils or NE. Further, administration of an NE inhibitor GW311616A significantly attenuates vascular leakage, leukocyte infiltration, and the expression of proinflammatory cytokines in the white adipose tissue from high-fat diet (HFD)-induced obese mice. Likewise, NE-deficient mice are resistant to HFD-induced vascular leakage in the heart. Together, NE regulates actomyosin cytoskeleton activity and VE-cadherin expression by activating PAR2 signaling in the endothelial cells, leading to increased vascular permeability and leukocyte extravasation. Hence, inhibition of NE is a potential approach to mitigate vascular injury and leukocyte infiltration in obesity-related systemic inflammation.

## 1. Introduction

The vascular endothelium is a monolayer of endothelial cells (ECs) between the bloodstream and the vessel wall. The endothelium is a barrier between blood and tissues and plays a vital role in controlling leukocyte adhesion and migration [1,2]. Injury of the endothelial layer leads to pathological changes such as vascular leakage, leukocyte infiltration, and inflammation in the vascular wall and adjacent tissues [3]. Neutrophils are the most abundant leukocytes with a short lifespan but play a critical role in tissue damage and inflammation during infection, as well as in various acute or chronic inflammatory diseases [4,5,6,7,8]. Activated neutrophils interact with vascular ECs, initiating an innate program of vascular adhesion and transendothelial migration at the site of inflammation. Neutrophils cause vascular injury and inflammation via neutrophil extracellular traps (NETs) and release intracellular contents, such as reactive oxidants and granule proteinases [9,10,11,12,13]. Neutrophil elastase (NE) is a neutrophil-specific serine protease secreted from the primary granules and is an important regulator and component of NETs [14,15]. As reported, neutrophils play a significant role in developing cardiovascular diseases in mouse models [16,17]. Studies from our group and others also revealed that deletion of NE impeded obesogenic-diet-induced inflammation in mouse adipose tissues, suggesting that NE mediates obesity-related inflammation and tissue damage [18,19,20,21]. However, further studies are needed to understand the molecular basis by which NE accelerates vascular injury and associated inflammation.

Increased vascular permeability allows small molecules and even whole cells, such as immune cells, in and out of the vessel at the site of inflammation. The features of increased endothelial permeability include the opening of intercellular junctions and the formation of gaps between endothelial cells [22]. VE-cadherin is one of the critical components of cell–cell junctions and plays a significant role in maintaining vascular endothelial barrier function [23,24,25]. VE-cadherin is associated with actin filament through interaction with actin-binding proteins, such as catenins [26,27,28]. Internalization and degradation of VE-cadherin promote the opening of the endothelial barrier [24,29]. Endothelial permeability is regulated by various factors, including VEGF and inflammatory mediators such as histidine, bradykinin, and thrombin [22,30,31]. Multiple studies revealed that signaling elements involved in actin filament formation and actomyosin contraction, such as G-protein-coupled receptors, small GTPase RhoA, ROCK, and myosin light chain kinase (MLCK), are the key components for the regulation of endothelial junction and permeability [31,32,33,34].

In this study, we attempted to investigate the direct effects of NE on cell permeability with cultured hECs. Our data revealed that NE activated PAR2, increased the formation of filament actin (F-actin), and decreased the expression of VE-cadherin at the intercellular junction. NE’s effects on F-actin formation and MLC phosphorylation were dependent on the activities of PAR2, ROCK, and MLCK. NE suppressed VE-cadherin gene expression by activating PAR2 but not ROCK in the endothelial cells. Yet, NE stimulated VE-cadherin protein degradation via MG132-sensitive proteasomes, and inhibition of either PAR2 or ROCK reversed NE-induced VE-cadherin protein degradation. Treatment of hECs with inhibitors of PAR2, ROCK, and MLCK impeded NE-induced endothelial permeability increase. Inhibition of NE or PAR2 also significantly reduced monocyte transmigration through endothelial monolayer pretreated with either neutrophils or purified NE. Thus, our study reveals that NE increases para-endothelial permeability by activating PAR2 and its downstream signaling via both ROCK-dependent and independent pathways in cultured hECs. In addition, we observed that administration of an orally active NE inhibitor alleviated high-fat-diet (HFD)-induced vascular leakage, leukocyte infiltration, and inflammation in white adipose tissue (WAT). Deletion of NE also protected mice from HFD-induced vascular leakage in the heart. Together, our data suggest that NE plays a pivotal role in obesity-related vascular endothelial injury and subsequent systemic inflammation.

## 2. Materials and Methods

### 2.1. Materials

Primary human arterial endothelial cells, culture media, and growth supplements were obtained from Lonza Bioscience (Portsmouth, NH, USA). Human Neutrophil elastase was purchased from Elastin Products Company (Owensville, MO, USA). MLCK inhibitor 18, proteasome inhibitor MG-132, and BCECF-AM were bought from Cayman chemicals (Ann Arbor, MI, USA). Neutrophil elastase inhibitor GW311616A was obtained from Axon (Reston, VA, USA). PET membrane transwell inserts (8 μm pore size) were purchased from Corning (New York, NY, USA). The phalloidin-iFluor 594 reagent was purchased from Abcam (Waltham, MA, USA). PAR2 (1-6) amide trifluroacetate salt was from Sigma Aldrich (St. Louis, MI, USA). Phospho-myosin light-chain kinase (p-MLC, Ser19), phospho-myosin phosphatase target subunit 1 (p-MYPT1, Thr853), β-catenin, HRP-conjugated goat anti-rabbit, and goat anti-mouse secondary antibodies, and Rho kinase (ROCK) inhibitor Y27632 were purchased from Cell Signaling (Beverly, MA, USA). VE-cadherin and p120-catenin antibodies, and HRP-conjugated donkey anti-goat secondary antibody were bought from Santa Cruz Biotechnology Inc. (Dallas, TX, USA). PAR2 antagonist I-191, Alexa-Fluor 568 goat anti-rabbit IgG and Alexa-Flour 488 chicken anti-goat IgG, transwell inserts (1.13 cm^2^ culture area, 0.4 μm pore size polycarbonate filter), and pierce TM ECL Western blotting chemiluminescence substrate solution were obtained from Thermo Scientific (Waltham, MA, USA).

### 2.2. Cell Culture

Primary human arterial endothelial cells (hECs) were obtained from Lonza Bioscience (Portsmouth, NH, USA) and cultured in endothelial cell growth basal (EBM) medium supplemented with EGM^TM^ Endothelial SingleQuots (0.04% hydrocortisone; 0.4% hFGF-B; 2% FBS; and 0.1% each of VEGF, R^3^-IGF-1, ascorbic acid, heparin, hEGF, and GA-1000), 10% fetal bovine serum, and 1% antibiotic solution at 37 °C in a 5% CO_2_ incubator. THP-1 monocytes were kindly provided by Dr. Andreea Bujor (Boston University) and grown in RPMI 1640 medium containing 10% fetal bovine serum and 1% antibiotic solution.

### 2.3. Experimental Animals

Male C57BL/6J and neutrophil elastase knockout (NEKO, B6.129 × 1-Elane^tm1Sds/J^) mice were obtained from The Jackson Laboratory and maintained under pathogen-free conditions in the vivarium of the at Boston University Medical Campus. Heterozygous NEKO mice were used for breeding to obtain NEKO homozygous and wild-type (WT) littermates for experiments. The mouse room was on a 12 h light/dark cycle, and standard mouse chow and water were available ad libitum. All procedures were performed according to animal research protocols approved by the Boston University Institutional Animal Care and Use Committee in compliance with the National Institutes of Health Animal Care and Use Guidelines. For experiments, 8-week-old mice were fed a normal chow diet (NCD; 2918, Teklad Animal Diets), a high-fat diet (HFD; 60 kcal% fat, D12492, Research Diets), or the HFD plus GW311616A (25 mg/kg HFD) for a period as designed for each experiment.

### 2.4. Evans Blue Vascular Permeability Test

Briefly, C57BL/6 mice (8-week-old males) were fed with either a chow diet or HFD (60 kcal% fat) for five weeks. Moreover, NEKO and WT littermates (7-week-old females) were fed the HFD for 10 weeks. Evans Blue (0.3% in sterile PBS with 3% bovine serum albumin, 5 μL/g body weight) was then administrated intravenously via retro-orbital injection to the mice anesthetized under 2–4% isoflurane plus O_2_ inhalation. Evans Blue dye was allowed to circulate in the blood for 2 h in mice. Then, mice were sacrificed with CO_2_ inhalation and immediately perfused with 50 mL PBS via left ventricle of the heart to remove dye in the blood vessels. Isolated epididymal fat pads or heart samples were washed and weighted before incubation in formamide at 65 °C for overnight to extract Evans Blue followed by reading optical absorbance at 620 nm with spectrophotometry. The amounts of Evans Blue in adipose tissues and heart were calculated according to a standard curve and the weight of the tissue used for extraction.

### 2.5. Isolation of the SVF and FACS Analysis

Anesthetized mice were perfused with PBS via left ventricles before perigonadal fat pads were excised and used for isolating SVF as described previously [18]. Mouse perigonadal fat pads were excised after the animals were perfused with 40 mL PBS via left ventricle and minced in PBS containing 1 mM CaCl_2_ and 0.5% BSA (Buffer I). The tissue suspensions were centrifuged at 500× *g* for 10 min to remove erythrocytes and free leukocytes. After centrifugation, the floating adipose tissue was incubated with 2 mg/mL collagenase in DMEM containing 0.5% BSA at 37 °C for 30 min with gentle shaking. Then, cell suspension was filtered through a 100 μm strainer and centrifuged at 500× *g* for 5 min to separate floating adipocytes from the SVF pellet. The pellet was resuspended in 0.5 mL red blood cell lysis buffer and washed with Buffer I. Zombie-Aqua stain was performed to differentiate live versus dead cells in each sample. The cells (1 × 10^6^) in SVF were blocked with 1 μg Fc receptor for 15 min at 4 °C before incubation with 0.5 mg fluorochrome-conjugated primary antibodies for 30 min at 4 °C in PBS containing 1 mM CaCl_2_ and 0.5% BSA. After staining with allophycocyanin (APC)-conjugated anti-Ly-6G antibody, phycoerythrin (PE)-conjugated anti-CD11b antibody, peridinin-chlorophyll-protein-complex (PerCP)-conjugated anti-CD45 antibody, FITC-conjugated anti-F4/80 antibody, and respective isotype controls, the cells were analyzed using BD LSRII flow cytometer, and data were analyzed using FlowJo software. Neutrophils were identified as CD45^+^CD11b^+^Ly-6G^+^ cells. Adipose tissue macrophages (ATMs) were identified as CD45^+^CD11b^high^F4/80^+^ cells. Anti-mouse CD45 PerCP-Cy5.5, anti-mouse Ly-6G-APC, anti-mouse CD11b-PE, anti-mouse F4/80-FITC, and the IgG controls were from eBioscience.

### 2.6. Real-Time Quantitative RT-PCR

Total RNA was isolated from adipose tissues using TRIzol reagent (Invitrogen, Carlsbad, CA, USA). RNA (1 μg/sample) was reverse-transcribed to cDNA with an iScript^TM^ cDNA synthesis kit (Bio-Rad, Hercules, CA, USA) in a final volume of 20 μL. Real-time qPCR was performed with SYBR Green JumpStart Taq Ready Mix (Sigma-Aldrich, St. Louis, MI, USA) using a ViiA 7 Real-Time PCR System (Life Technologies, Waltham, MA, USA). 36b4 was amplified as an internal control. The results are expressed as the fold change in mRNA levels relative to the expression of 36b4 in the same samples and were calculated with the following formula based on two replications of each cycle: fold change = [efficiency (c–t) of target gene/efficiency (c–t) of 36b4 mRNA], where c represents the cycle threshold (Ct) for the target gene or 36b4 in the experimental control samples, and t represents Ct for the target gene or 36b4 in the test samples.

### 2.7. Mouse and Human PCR Primers

m36B4: Forward 5′-TCGGGTCCTAGACCAGTGTTC-3′, Reverse 5′-AGATTCGGGATATGCTGTTGGC-3′;

mTNFα: Forward, 5′- CCAGTGTGGGAAGCTGTCTT -3′ Reverse, 5′-AAGCAAAAGAGGAGGCAACA-3′;

mIL1β: Forward 5′-TGGAAAAGCGGTTTGTCT-3′, Reverse 5′-ATAAATAGGTAAGTGGTTGCC-3′;

mIL-6: Forward, 5′-TACCACTTCACAAGTCGGAGGC-3′, Reverse 5′-CTGCAAGTGCATCATCGTTGTTC-3′;

hVE-cadherin: Forward 5′-GAAGCCTCTGATTGGCACAGTG-3′, Reverse 5′-TTTTGTGACTCGGAAGAACTGGC-3′;

h36B4: Forward 5′-CGACCTGGAAGTCCAACTAC-3′ Reverse 5′-ATCTGCTGCATCTGCTTG-3′.

### 2.8. Immunofluorescence and F-Actin Phalloidin Staining

hECs were fixed with 4% paraformaldehyde, permeabilized with PBST (0.3% Triton X-100 in PBS), and blocked with SEA BLOCK buffer (Thermo Scientific, Waltham, MA, USA) for 1 h at room temperature. Subsequently, the cells were incubated with appropriate primary antibody at 4 °C overnight and washed three times with PBST for 20 min. The cells were then incubated with corresponding Alexa-fluor-conjugated secondary antibodies for 2 h at room temperature in the dark. For cell surface PAR2 immunofluorescent staining with non-permeabilized cells, PBS containing 0.5% goat serum was used for both primary and secondary antibody staining and washing. For F-actin staining, cells were stained with Phalloidin-iFluor 594 reagent (Abcam, ab176757) for 30 min at room temperature and washed with PBS for 20 min. Air-dried coverslips were mounted using ProLong Gold anti-fade agent with DAPI (Invitrogen), and images were captured using an inverted phase contrast microscope (Keyence BZ-9000E, Magnification X20/0.75 NA) in florescence mode.

### 2.9. Immunoblotting

Total protein was extracted from cells using lysis buffer containing 50 mM HEPES (pH 7.4), 137 mM NaCl, 5 mM sodium pyrophosphate, 5 mM β-glycerophosphate, 10 mM sodium fluoride, 2 mM EDTA, 2 mM Na_3_VO_4_, 1 mM PMSF, 10 μg/mL aprotinin, 10 μg/mL leupeptin, 10 nM calyculin A, and 1% Triton X-100, as described previously [35]. Total cell lysates were boiled in the sample buffer for 5 min, and 30 μg of protein was resolved with SDS/PAGE and electro-transferred to nitrocellulose membranes. After blocking with 4% BSA for 30 min, the membrane was probed with appropriate primary antibodies against VE-cadherin, p-MYPT1 (Thr853), MYPT1, p-MLC (Ser 19), MLC, β-catenin, and p120- catenin at 4 °C overnight. The membranes were washed with TBST and incubated in the horseradish-peroxidase-conjugated secondary antibodies for 1 h at room temperature. The specific protein bands were detected using Pierce^TM^ ECL Western Blotting Chemiluminescence substrate solution (Thermo Scientific, Waltham, MA, USA).

### 2.10. Percoll Gradient Purification of Neutrophils

Mouse neutrophils were isolated, as described, by harvesting bone marrow from femur and the tibia of C57BL/6J mice [36]. After bone marrow cells were washed twice with PBS, neutrophils were purified by continuous Percoll gradient centrifugation. Briefly, percoll gradient solution was prepared by layering 2 mL each of the 62, 55, and 50% percoll solutions on the top of 81% percoll solution. Bone marrow cell suspension was added on the top of gradient and centrifuged at 1600× *g* for 30 min with no brake at 4 °C. The cell layer was collected from between 81 and 62% percoll layer and washed with PBS twice at 500× *g* for 5 min. Red blood cells were lysed with ACK lysis buffer (Thermo Fisher Scientific), and the cells were again washed with PBS and resuspended in cell media. The purity of the neutrophil cells was greater than 95%.

### 2.11. Lucifer Yellow Permeability Study

Permeability across endothelial cell monolayers was measured using transwell inserts (1.13 cm^2^ culture area, 0.4 μm pore size polycarbonate filter, Thermo Scientific, Waltham, MA, USA) as described previously [33]. Briefly, the inserts were first coated with collagen type 1 overnight at 37 °C for cell attachment. hECs (1 × 10^5^ cells/mL) were seeded on the transwell inserts coated with collagen type-I in 12-well plates and incubated for 48 h until confluency. The cells were then preincubated with various inhibitors for 20 min and then treated with NE, PAR2 agonist, or variant inhibitors for 16 h. Then, Lucifer Yellow (1 mM) was added to the upper compartment of the transwell inserts for 1 h. The amount of Lucifer Yellow that penetrated through the cell monolayer into the lower compartment was measured using a multimode microplate reader (485 nm excitation and 530 nm emission wavelength). The fluorescent intensity from the transwell without seeding hECs was set as 100%, and the maximum permeability in the percentage of control (Fluorescent intensity of stimulated cells divided by control) are presented.

### 2.12. Monocyte Transmigration

THP-1 monocyte transmigration through endothelial cell monolayer was performed as described previously [37]. Briefly, hECs were grown to confluence on type I collagen-coated transwell inserts (0.9 cm^2^ culture area, 8 μm pore size polyethylene terephthalate membrane, Corning, New York, NY, USA) before treatment with NE, variant inhibitors, or isolated neutrophils (1 × 10^6^ cells/well) for 16 h. Then, hECs were washed with blank media to remove remaining reagents or neutrophils before adding BCECF-AM-labelled THP-1 cells (1 × 10^5^) for transmigration for two hours with neutrophil-pretreated hECs and for six hours with NE-pretreated cells. THP-1 cells were labeled with BCECF-AM (Abcam, Waltham, MA, USA) at 1 nM/10^6^ THP1 cells for one hour at 37 °C and washed with PBS before being used for transmigration assay. After transmigration, cells from the apical side were removed with cotton swab, and membrane was cut, fixed, and placed in microscopic slides. THP-1 cells transmigrated through the hEC monolayer on the bottom side of the membrane were observed under an inverted phase contrast microscope (Keyence BZ-9000E, Magnification X20/0.75NA), and fluorescence images (Excitation 488 nm, Emission 520 nm) were captured. For quantification, monocytes were counted in five random microscopic fields per well, and the mean number of cells per field was calculated.

### 2.13. Statistical Analysis

Significant differences among groups were determined by one-way analysis of variance (ANOVA) followed by Tukey’s multiple comparison test using the software GraphPad Prism Version 9.3.0 (GraphPad Software, Inc., La Jolla, CA, USA). For the mouse studies, evaluations were performed by two-way ANOVA and unpaired Student’s *t*-test. All results are presented as mean ± SD. The threshold for *p*-value significance was set at *p* < 0.05.

## 3. Results

### 3.1. NE Regulated Vascular Endothelial Cell Permeability, Actomyosin Cytoskeleton, and the Expression of Cell–Cell Junction Molecule VE-Cadherin

**Figure 1:**
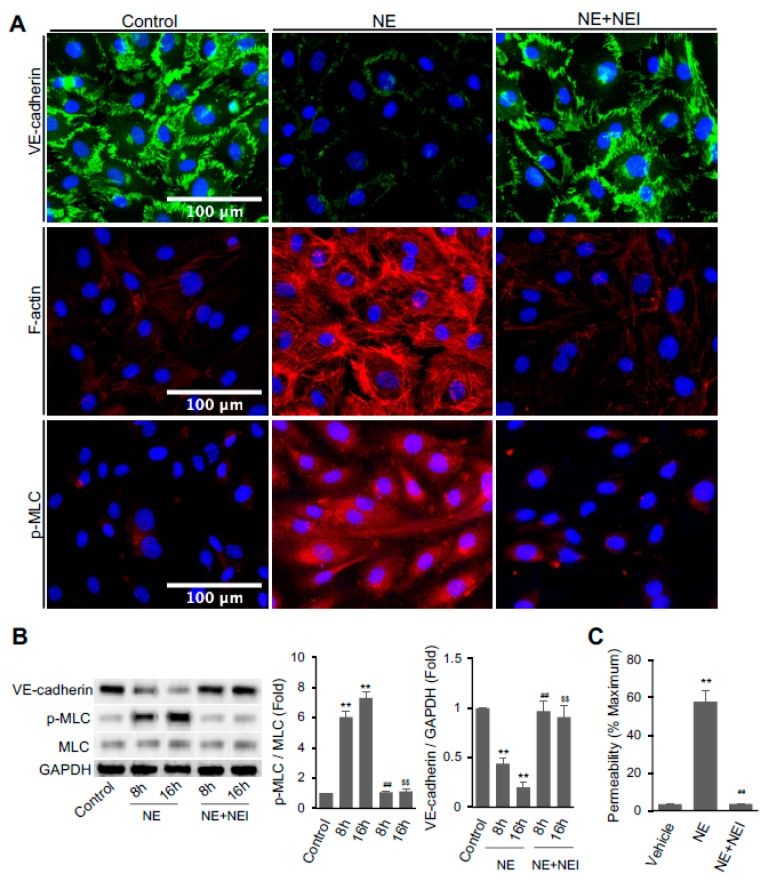
Neutrophil elastase (NE) decreased VE-cadherin protein and increased F-actin formation, MLC phosphorylation, and permeability in hECs. (**A**) Confluent hECs were treated with the vehicle or 20 nM NE in the presence or absence of NE inhibitor GW311616A (NEI, 1 μM) for 16 h. Cells were fixed and fluorescence labeled with antibodies against VE-cadherin (Alexa-Fluor 488, green, top panel) and p-MLC (pSer19, Alexa-Fluor 568, red, lower panel) or phalloidin (ifluor 594, red, middle panel) to analyze the distribution of VE-cadherin, p-MLC, and F-actin. Images were taken at 20x magnification (scale bar, 100 μm). Representative images were chosen from three independent experiments. (**B**) Western blot analysis of VE-cadherin, p-MLC, total MLC, and GAPDH in hECs treated with or without NE for 8 or 16 h in the presence or absence of NEI (1 μM). (**C**) hEC permeability assay Lucifer Yellow dye. Graph shows endothelial cell permeability after 16 h of NE treatment. (**B**,**C**) All data are presented as mean ± SD of three independent experiments. ** *p* < 0.01 vs. vehicle control group, ^##^ *p* < 0.01 vs. NE-8h group, and ^$$^ *p* < 0.01 vs. NE-16h group. NE: neutrophil elastase (20 nM), NEI: neutrophil elastase inhibitor. Control vehicle used for the experiment was PBS containing 0.5 mM sodium acetate that was used for dissolving purified NE. See also Figure S1.

We examined if NE directly affects endothelial functions. hECs were treated with or without NE (20 nM) for 16 h before immunofluorescence staining to detect VE-cadherin distribution, F-actin formation, and MLC phosphorylation. hEC monolayer treated with the control vehicle showed intact well-established VE-cadherin at the cell–cell junctions. Upon treatment with NE for 16 h, VE-cadherin at the cell boundary was dramatically reduced (Figure 1A, top panel). However, VE-cadherin remained intact at the intracellular junctions of hECs pretreated with 1 μM GW311616A, a NE inhibitor (NEI) [38]. Actin filament formation and phosphorylation of myosin light chain (p-MLC) play a crucial role in endothelial barrier disruption. We also applied phalloidin-iFluor 594 reagent and antibody against p-MLC (Ser19) to visualize the effects of NE on F-actin distribution and phosphorylation status of MLC in hECs, respectively. Treatment of hECs with NE markedly enhanced the formation of actin stress fibers, whereas NEI prevented NE’s effect (Figure 1A, middle panel). Similarly, NE increased the staining of p-MLC in hECs, but NEI diminished the phosphorylation (Figure 1A, lower panel).

We also performed a study to examine the dose-response characteristics for both NE and its inhibitor GW311616A with cultured hECs. Our data revealed that purified NE (5, 10, and 20 nM) had dose-responsive effects on reducing cell surface VE-cadherin and enhancing F-actin formation (Figure S1). NE showed minimal effects at 5 nM concentration and apparent effects at 10 nM, reaching the maximal effect at 20 nM. GW311616A inhibited NE (20 nM)-induced loss of VE-cadherin at 200 nM and completely reversed NE’s effect at 1 μM. However, GW311616A effectively blocked NE-induced F-actin formation even at the concentration of 100 nM.

Western blot analysis revealed that total VE-cadherin protein expression in hECs was significantly reduced after treatment with NE for 8 or 16 h (Figure 1B). Treatment with NEI completely reversed the effect of NE on VE-cadherin expression. hECs treated with NE had a significant increase in phosphorylation of MLC compared to control cells. Again, NEI significantly reduced the ratio of p-MLC over total MLC to a level comparable to the control group.

We performed transwell assay using Lucifer Yellow dye to determine whether the NE-induced changes in endothelial cytoskeleton and disruption of adhesion molecule VE-cadherin are involved in endothelial cell permeability. As per the results presented in Figure 1C, the treatment of confluent hEC monolayer with NE for 16 h significantly increased the permeability from 57.84 ± 5.87% to vehicle-treated cells (3.55 ± 0.20%). Again, NEI pretreatment completely prevented NE-induced hEC leakage.

### 3.2. NE Regulated hEC Permeability through Activating Protease-Activated Receptor 2 (PAR2)

**Figure 2:**
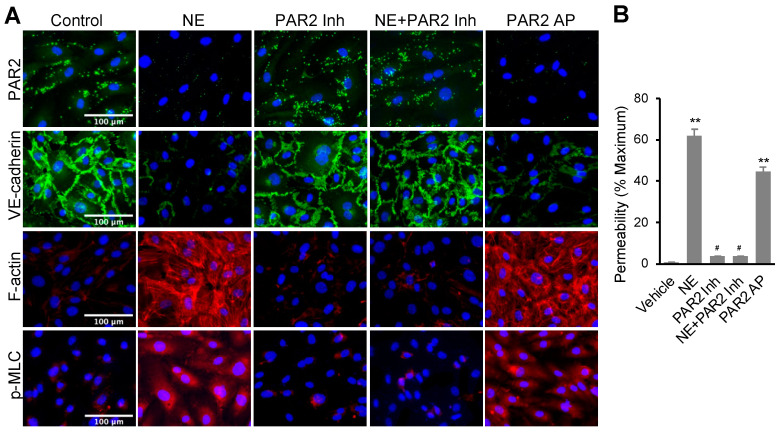
NE regulated VE-cadherin, actomyosin cytoskeleton, and permeability in hECs through activating protease-activated receptor 2 (PAR2). (**A**) Immunofluorescence images of PAR2 (green), VE-cadherin (green), F-actin (red), or p-MLC (pSer19, red) and nuclei counterstaining DAPI (blue). hEC cells were treated with NE (20 nM) or PAR2 agonist (PAR2-AP, 7.5 μM) in the presence or absence of PAR2 inhibitor (1 μM) for 16 h. Images were taken at 20x using a fluorescence microscope; scale bar: 100 μm. (**B**) Graph showing percentage permeability of endothelial monolayer treated with 20 nM NE, 7.5 μM PAR2 agonist, and 1 μM PAR2 inhibitor. Permeability assay was carried out with Lucifer Yellow dye. All data are expressed as mean ± SD of three independent experiments. ** *p* < 0.01 vs. vehicle control group, ^#^ *p* < 0.05 vs. NE group. NE: neutrophil elastase, PAR2 Inh: protease-activated receptor 2 inhibitor (I191), PAR2 AP: protease-activated receptor 2 agonist (PAR2 (I-6) amide trifluroacetate salt). See also Figures S2–S4.

NE is known to activate multiple signal pathways including Toll-like receptor 4 (TLR4), NFκB, and p42/p44 MAP kinase [39,40,41,42,43,44,45]. We examined if inhibitors of those signal pathways regulate NE’s effects on VE-cadherin and F-actin. As shown in Figure S2, hECs treated with TAK-242 (TLR4 inhibitor), BAY-117082 (NFκB inhibitor), and U0126 (MEK inhibitor) did not alter NE-induced VE-cadherin loss and F-actin formation, suggesting the NE’s effects are independent of those pathways. We also accessed the effect of NE treatment on PAR2 signaling in hECs. Immunofluorescence staining of cell surface PAR2 was performed with hECs. PAR2 (I-6) amide trifluoroacetate (LSIGKV-NH_2_) and non-peptide small molecule I191 were applied as a PAR2 agonist (PAR2-AP) and a non-competitive binding PAR2 inhibitor, respectively, in this study [46,47]. As shown in Figure 2A (upper panel), hECs treated with NE (20 nM) or PAR2-AP (7.5 μM) for 16 h significantly decreased cell surface expression of PAR2. Conversely, I191 (1 μM) fully impeded NE-induced PAR2 internalization. PAR2 agonist reduced VE-cadherin expression as found in NE-treated cells. PAR2 antagonist blocked NE-induced VE-cadherin decline in hECs, although the antagonist alone had no significant effect on the adhesion molecule (Figure 2A, second panel). Further, the PAR2 agonist had similar effects as NE with increased F-actin formation and p-MLC in hECs compared to the control group. In contrast, pretreatment of the cells with the PAR2 antagonist obstructed the impact of NE (Figure 2A, third and fourth panels). As expected, cells pretreated with PAR2 antagonist alone showed no effect on F-actin and p-MLC. To further validate our findings, we also applied the known PAR2 activator trypsin and a different PAR2 antagonist, ENMD1068, to our study. Treatment of hECs with trypsin (100 nM) for 16 h, similar to the effect of NE, induced the loss of PAR2 and VE-cadherin on the cell surface and the increase in F-actin formation, while ENMD1068 blocked the impact of both NE and trypsin (Figure S3). Together, our data suggest that NE activates PAR2, which is required for NE’s regulation of F-actin, p-MLC, and VE-cadherin in hECs.

We compared the effects of NE and PAR2 regulators on endothelial cell permeability. In this experiment, confluent hEC monolayers on collagen-coated transwell were treated with PAR2-AP (7.5 μM) or NE (20 nM) for 16 h. The basal hEC permeability was 3.74 ± 0.11%. Both NE and PAR2-AP alone induced a significant increase in hEC permeability to 62.03 ± 3.04% and 44.79 ± 2.16%, respectively (Figure 2B). PAR2 inhibitor I191 (1 μM) alone did not affect basal permeability. Still, it significantly inhibited NE-induced Lucifer Yellow dye leakage through hEC monolayer, suggesting NE’s effect on permeability is dependent on the activation of PAR2.

### 3.3. NE’s Effects on hEC Permeability, F-Actin Formation, MLC Phosphorylation, and VE-Cadherin Expression Were Mediated via PAR2 Downstream Signaling Pathway

**Figure 3:**
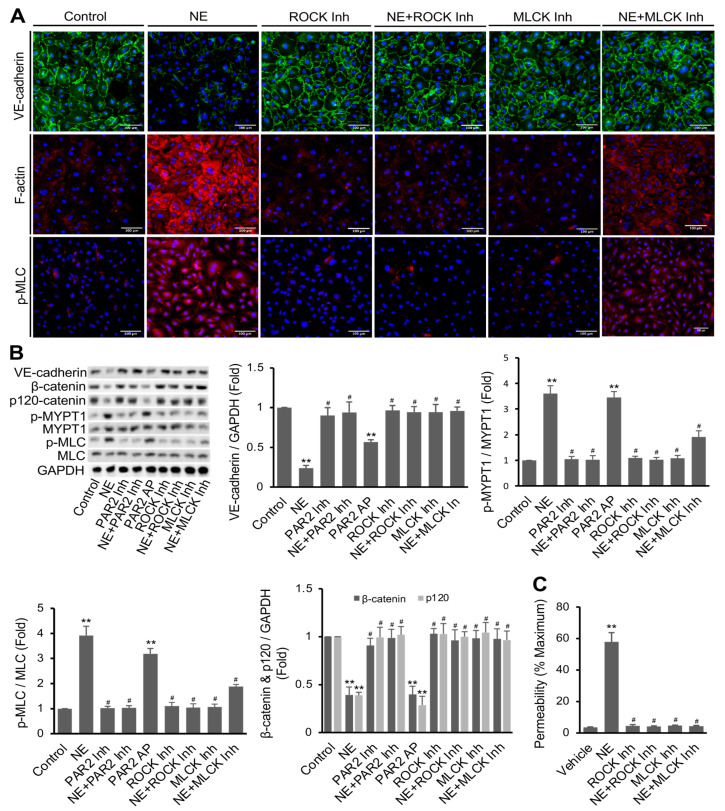
Regulation of NE’s effects by PAR2 downstream signaling components ROCK and MLCK. (**A**) hECs were treated with ROCK inhibitor (Y27632, 5 μM) and MLCK inhibitor (peptide 18, 5 μM) with or without NE (20 nM) for 16 h. Images (20x) were taken after immunofluorescence staining of VE-cadherin (green) and p-MLC (pSer19, red), F-actin staining with phalloidin (red), and nuclei staining with DAPI (blue). Images are representatives of three independent experiments (scale bar, 100 μm). (**B**) Western blot analysis confirming the changes of VE-cadherin, β-catenin, p120-catenin, p-MYPT1 (pThr853), and p-MLC (pSer19) in hECs. GAPDH was used as an internal control for protein loading. (**C**) Confluent hECs on transwells (with 0.4 μm pore size) were treated with or without NE (20 nM) in the presence or absence of ROCK or MLCK inhibitor for 16 h. Lucifer Yellow was used to measure cell permeability, and the results were expressed as percentage of control (transwells without cells). All data are expressed as mean ± SD of three independent experiments. ** *p* < 0.01 vs. vehicle control group, ^#^ *p* < 0.05 vs. NE group. NE: neutrophil elastase, ROCK Inh: ROCK inhibitor, MLCK Inh: MLCK inhibitor. See also Figure S4.

We further examined whether NE’s effects on F-actin, p-MLC, VE-cadherin, and permeability in hECs are dependent on the activation of PAR2 downstream signaling components such as myosin light chain kinase (MLCK) and Rho kinase (ROCK) [48]. In this study, hECs were pretreated with the ROCK1/2 inhibitor Y-27632 (5 μM) and MLCK inhibitor peptide-18 (5 μM) for 20 min prior to adding NE (20 nM). Control vehicle-treated cells showed a well-defined VE-cadherin expression and low basal levels of F-actin fibers and p-MLC. Treatment of hECs with Y-27632 or peptide-18 alone did not show a noticeable effect on basal VE-cadherin expression but significantly reversed NE-induced diminution of VE-cadherin (Figure 3A, top panel). Yet, NE-induced F-actin formation and p-MLC were completely repressed by ROCK inhibitor and partially reduced by MLCK inhibitor (Figure 3A, middle and lower panels). Western blot analysis was performed to quantify the protein levels of VE-cadherin, p-MLC, and p-MYPT1, a direct downstream target of ROCK1/2 [49]. hECs treated with NE showed a decreased protein expression of VE-cadherin at 16 h. NE caused a 3.92 ± 0.36-fold increase in p-MLC and a 3.60 ± 0.30-fold increase in p-MYPT1 compared to vehicle-treated cells (Figure 3C). Likewise, PAR2-AP also increased p-MLC (3.19 ± 0.21-fold) and p-MYPT1 (3.45 ± 0.11-fold) at a statistically significant level (*p* < 0.001). Pretreatment of the cells with PAR2 antagonist or ROCK inhibitor almost completely blocked NE-induced MLC and MYPT1 phosphorylation, whereas MLCK inhibitor also significantly reduced NE’s effects. Inhibition of ROCK or MLCK alone did not affect basal permeability with confluent hECs. However, pretreatment of the cells with Y-27632 or peptide-18 significantly inhibited the NE-mediated increase in hEC permeability (Figure 3C).

We also performed experiments to determine the effects of ROCK and MLCK inhibitors on PAR2 agonist PAR2-AP-induced endothelial permeability, F-actin distribution, and phosphorylation of MLC. Both immunofluorescence staining and Western blot analysis revealed that the PAR2 agonist caused VE-cadherin degradation and increased MLC phosphorylation compared to untreated cells. Figure S4A show that either ROCK inhibitor Y-27632 or MLCK inhibitor peptide-18 completely attenuated PAR2-AP-induced changes in VE-cadherin expression in hECs. Notably, PAR2-AP-induced phosphorylation of MLC was entirely blocked by the ROCK inhibitor but partially inhibited by the MLCK inhibitor (Figure S4B). Yet, PAR2-AP significantly stimulated hEC permeability, and this effect was impeded by inhibition of either ROCK or MLCK (Figure S4C). Together, our data suggest that activation of PAR2, similar to NE treatment, reduces VE-cadherin protein expression at the intercellular junction, stimulates F-actin formation and MLC phosphorylation, and increases cell permeability in cultured hECs. Inhibition of either ROCK or MLCK hampers the effects of PAR2 activation.

### 3.4. NE Induced VE-Cadherin Protein Degradation and Repressed VE-Cadherin Gene Expression

**Figure 4:**
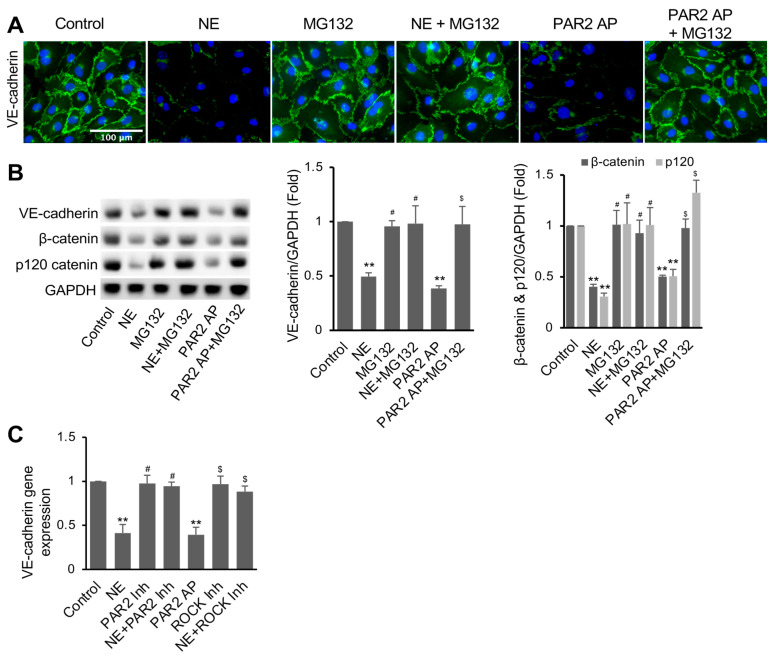
Inhibition of proteasome activity reversed NE-induced VE-cadherin degradation and permeability in hECs. Cells were treated with or without proteasome inhibitor MG132 (5 μM) for 2 h before adding NE (20 nM) or PAR2 agonist (Par2-AP, 7.5 μM) for 16 h, as indicated. Then, cells were used for immunofluorescence imaging of VE-cadherin and Western blot analysis. (**A**) VE-cadherin fluorescence images (green) are representative of three independent experiments (scale bar, 100 μm). (**B**) Western blot images and quantification showing VE-cadherin, β-catenin, and p120-catenin in hECs. (**C**) Gene expression of VE-cadherin in hECs treated with NE (20 nM) or PAR2 agonist (Par2-AP, 7.5 μM) in the presence or absence of ROCK inhibitor (Y27632, 5 μM) for 16 h. RT-PCR was used for quantifying gene expression levels of VE-cadherin normalized to 36B4 for each sample. All data are expressed as mean ± SD of three independent experiments. ** *p* < 0.01 vs. vehicle control, ^#^ *p* < 0.05 vs. NE group, ^$^ *p* < 0.05 vs. PAR2-AP. PAR2-AP: PAR2 agonist (PAR2 (I-6) amide trifluroacetate salt) (7.5 μM), ROCK Inh: ROCK inhibitor. See also Figure S4.

Since protein levels of VE-cadherin were decreased in hECs treated with NE or the PAR2 agonist, we further tested if proteasome activity is involved in the degradation of VE-cadherin degradation. Remarkably, pretreatment of hECs with proteasome inhibitor MG132 (5 μM) for 2 h before adding NE or PAR2-AP for 16 h significantly inhibited NE- or PAR2-AP-induced VE-cadherin degradation, as shown with both immunofluorescence staining and Western blotting (Figure 4A,B). Remarkably, NE and PAR2-AP lowered the protein levels of β-catenin and p120-catenin; both are actin-binding and VE-cadherin-interacting proteins in the endothelial cells. Surprisingly, pretreatment of the cells with MG132 inhibited NE- and PAR2-AP-induced β-catenin and p120-catenin degradation (Figure 4B). Our data suggest a crucial role of proteasome-mediated degradation of cadherin-binding p120-catenin and β-catenin in maintaining cell surface VE-cadherin in hECs.

It has been suggested that activation of the PAR2 pathway suppresses VE-cadherin gene expression [50,51]. We also examined the effects of NE and PAR2 signaling regulators on VE-cadherin gene expression in hECs. Treatment of hECs with NE for 16 h, similar to PAR2-AP, dramatically suppressed VE-cadherin mRNA levels, whereas inhibition of PAR2 with PAR2 inhibitor I191 (1 μM) reversed the inhibitory effect of NE (Figure 4C). However, ROCK inhibitor Y-27632 failed to reverse NE’s effect on VE-cadherin gene expression. Together, our data suggest that the NE-PAR2 signaling reduces cell surface VE-cadherin levels through ROCK-cytoskeletal-dependent VE-cadherin protein degradation and ROCK-independent transcriptional repression.

### 3.5. NE Stimulated Monocyte Transendothelial Migration Dependent on the Activation of the PAR2 Signal Pathway

**Figure 5:**
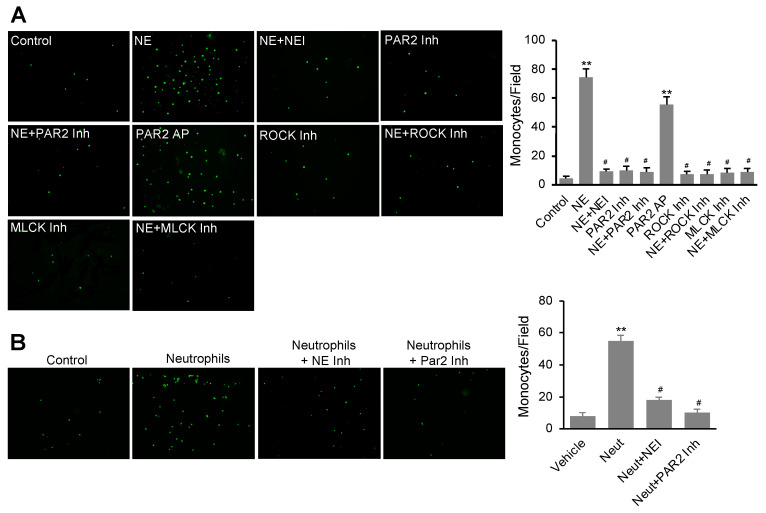
Interaction of endothelial cells with NE or isolated neutrophils stimulated monocyte transendothelial migration via activation of the PAR2 pathway. (**A**) Confluent hECs grown on collagen-coated transwell inserts (8 μm pore size) were treated with NE (20 nM) or PAR2 agonist (PAR2-AP, 7.5 μM) in the presence or absence of NE inhibitor (NEI, 1 μM), PAR2 inhibitor (PAR2 Inh, 1 μM), ROCK inhibitor (ROCK Inh, 5 μM), and MLCK inhibitor (MLCK Inh, 5 μM) for 16 h. hECs were washed with normal media before adding BCECF-AM labeled THP-1 cells (1 × 10^5^) for 6 h. (**B**) Confluent hECs on transwell inserts were treated with freshly isolated neutrophils (1 × 10^6^ cells/well) for 16 h in the presence or absence of NE inhibitor (NEI, 1 μM) or PAR2 inhibitor (I191, 1 μM). hEC monolayer on the top chamber was washed with cell media to remove neutrophils. Then, BCECF-AM labeled THP-1 cells (1 × 10^5^ cells/well) were used to assess transmigration for 2 h. Representative images show fluorescence-labeled THP-1 cells transmigrated through the hEC monolayer were captured (**A**,**B**). For quantification, monocytes were counted in five random microscopic fields per well. The values are expressed as mean ± SD of three independent experiments. Top panel: ** *p* < 0.01 vs. vehicle control group, ^#^ *p* < 0.05 vs. NE group; lower panel: ** *p* < 0.01 vs. vehicle control group, ^#^ *p* < 0.05 vs. neutrophil (Neut) group.

We further evaluated the effects of NE and PAR-AP on the permeability of hEC monolayer to THP-1 cells (a monocyte cell line) using a transmigration assay. In this study, confluent hECs on the transwell were treated with or without NE or PAR2-AP for 16 h before washing out NE and PAR2-AP and adding THP-1 cells for 4 h to assess transmigration as described in the Materials and Methods section. As shown in Figure 5A, both NE and PAR-AP alone stimulated a significant increase in THP1 cell transmigration through the hEC monolayer. Interestingly, pretreatment of the cells with NEI (GW311616A), ROCK inhibitor Y-27632, or MLCK inhibitor peptide-18 suppressed NE-induced transmigration of the monocytes. Similarly, the ROCK and MLCK inhibitors also impeded PAR2-AP-stimulated monocyte migration. Our data suggest that NE accelerates monocyte transmigration through the endothelial monolayer by activating the PAR2 pathway in hECs.

We performed a further experiment to evaluate whether inhibition of NE affects monocyte transmigration through endothelial monolayer pretreated with purified neutrophils. As shown in Figure 5B, pretreatment of confluent hECs with neutrophils isolated from the bone marrows of C57Bl/6J mice overnight increased transmigration of fluorescent-labeled THP1 cells. However, inhibition of NE or PAR2 during the co-culture of hECs and neutrophils significantly blocked subsequent THP1 cell transmigration, suggesting that the activity of NE released from neutrophils and PAR2 signaling in hECs are required for THP1 cell transmigration induced by neutrophil–endothelial cell interaction.

### 3.6. Inhibition of NE Attenuated HFD-Induced Vascular Leakage in White Adipose Tissue (WAT) and Heart, as Well as Leukocyte Infiltration and Inflammation

**Figure 6:**
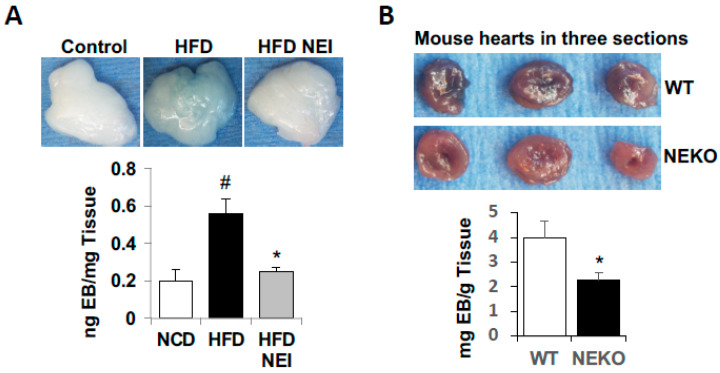
Inhibition of NE attenuated HFD-feeding-induced vascular leakage in WAT and heart. (**A**) C57BL/6J mice (8-week-old males) were fed either a standard chow diet, HFD (60% kcal of fat), or HFD plus NEI (GW311616A, 25 mg/kg HFD) for 5 weeks. (**B**) NEKO and WT controls (7-week-old, females) were fed with 60% HFD for 10 weeks. Then, at the end of feeding study, mice were injected with Evans Blue-albumin (EB, intravenous retro-orbital injection) for 2 h before being perfused with PBS to remove the dye in the blood vessels. Evans Blue leakage into epididymal fat (**A**) and heart (**B**) was quantified as described in the Materials and Methods section. (**A**,**B**) Top panels show representative WAT and heart images; lower panels show quantification of Evens Blue-albumin dye. (**A**) Data are presented as mean ± SD, *n* = 4 in each group. ^#^ *p* < 0.05, chow versus HFD; * *p* < 0.05, HFD vs. HFD plus NEI. (**B**) Data are presented as mean ± SD, *n* = 5/group, * *p* < 0.05, NEKO vs. WT.

Neutrophils are involved in tissue damage and inflammation in diet-induced obesity. Here, we applied both short-term and long-term diet-induced obese mouse models to assess the effects of inhibition of NE with orally active GW311616A on HFD-induced vascular leakage and inflammatory cell infiltration in WAT. For the short-term experiment, C57BL/6J mice were fed a normal chow diet (NCD), HFD (60% kcal of fat), or HFD plus GW311616A (25 mg GW311616A/kg food) for five weeks. Then, vascular leakage was assessed in vivo with Evans Blue-albumin dye, as described in the Materials and Methods section. Mice fed the HFD alone significantly increased Evans Blue dye in the epididymal WAT compared to NCD-fed control mice (Figure 6A). However, administration of GW311616A diminished HFD-induced Evans Blue-albumin dye leaking into the WAT, suggesting inhibition of NE protects mice from HFD-induced vascular injury and permeability in vivo.

We also compared the responses of NE knockout (NEKO) mice and their wild-type (WT) controls to HFD-feeding-induced vascular leakage in the heart. In this experiment, both NEKO and WT mice were challenged with HFD (60% Kcal of fat) for 10 weeks before permeability test with Evans Blue-albumin dye. As shown in Figure 6B, Evans Blue-albumin leaking into the heart was significantly higher in WT mice than NEKO mice, further supporting the role of NE in obesity-related vascular leakage.

**Figure 7:**
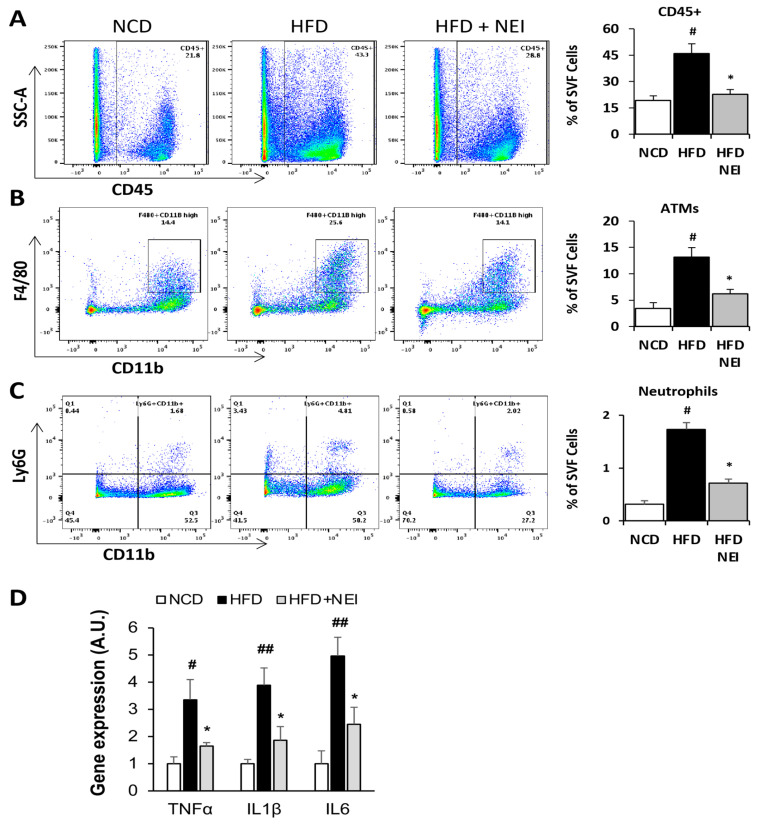
Inhibition of NE attenuated HFD feeding-induced leukocyte infiltration and pro-inflammatory cytokine expression in WAT. C57BL/6 mice (8-week-old males) were fed with either a standard chow diet, an HFD (60% kcal of fat), or HFD plus NEI (GW311616A, 25 mg/kg HFD) for 14 weeks before being perfused with PBS to remove blood cells in the circulation. SVF cells were isolated from eWAT for flow cytometry analyses of total CD45^+^ immune cells (**A**), CD45^+^CD11b^high^F4/80^+^ adipose tissue macrophages (ATMs) (**B**), and CD45^+^CD11b^+^Ly6G^+^ neutrophils (**C**). Total RNA samples from eWAT were used for RT-PCR to quantify mRNA expression levels of IL-1β, TNFα, and IL6 (**D**). The expression levels were normalized against the 36B4 reference gene. All the values are expressed as mean ± SD, *n* = 5 or 6 in each group of mice. ^#^ *p* < 0.05, ^##^ *p* < 0.01, chow versus HFD; * *p* < 0.05, HFD vs. HFD plus NEI. See also Figure S5.

For the long-term feeding experiment, three groups of male C57BL/6J mice were fed identical diets and the NE inhibitor as described in the short-term feeding study but for 14 weeks. Mice were then systemically perfused with phosphate-buffered saline (PBS) to remove circulating blood cells before isolating stromal vascular fractions (SVF) from epididymal WAT. We quantified adipose tissue macrophages (ATMs) and neutrophils in SVF cells with flow cytometry analysis (see Figure S5 for the gating strategy). HFD feeding increased total CD45^+^ immune cells, CD45^+^CD11b^+^Ly6G^+^ neutrophils, and CD45^+^CD11b^high^F4/80^+^ ATMs in SVF, while inhibition of NE significantly reduced HFD-induced accumulation of those immune cells in WAT (Figure 7A–C). Further, we compared expression levels of pro-inflammatory genes in WAT samples. As shown in Figure 7D, gene expression levels of IL1β, TNFα, and IL6 were all significantly increased in WAT from HFD-fed mice compared to the NCD control group. Again, feeding mice with the NE inhibitor and HFD significantly diminished gene expression of those inflammatory factors. Our data confirmed that inhibition of NE impedes HFD-induced leukocyte infiltration and inflammation in WAT in obese mice.

## 4. Discussion

Neutrophils play a vital role in the regulation of vascular permeability [12,52]. In response to inflammatory stimulation, neutrophils interact with the vascular wall in the inflamed tissues, resulting in vascular injury and permeability increase to macromolecules and immune cells, and subsequent pathological tissue damage. A better knowledge of the molecular mechanisms controlling these processes might thus help design new therapeutic strategies for the treatment of inflammatory diseases. Activated neutrophils release intracellular proteases such as NE via degranulation and netosis [53,54]. In this study, we examined the direct effects of neutrophil-specific protease NE on human vascular ECs. We observed that NE at a low level (20 nM) strikingly increased Lucifer Yellow permeability and monocyte transmigration through hEC monolayer in vitro. NE inhibitor GW311616A completely blocked NE’s effects on hEC permeabilities. Co-culture of hEC monolayer with isolated BM neutrophils also induced monocyte transmigration. Yet, inhibition of either NE or PAR2 significantly reduced the effects of neutrophil pretreatment on leukocyte transmigration, supporting the role of the endogenous elastase from neutrophils and its downstream PAR2 signaling on endothelial permeability and leukocyte transmigration. Further, we observed that administration of the orally active NE inhibitor impeded vascular leakage, leukocyte infiltration, and expression of inflammatory cytokines in the white adipose tissue in mice fed an HFD. Similarly, our study also revealed that mice lacking NE were resistant to HFD-induced vascular leakage in the heart. Thus, NE plays a significant role in regulating vascular endothelial permeability in vitro and obesity-related vascular injury and inflammatory infiltration in vivo. Over the last three decades, studies have linked obesity to increased inflammation in multiple tissues, such as white adipose tissues [55,56,57,58,59,60,61]. Neutrophils are the first line of innate immune cells that respond to infection and inflammation. Previously, we reported that neutrophil number and NE expression are increased in mice at the early stage of HFD feeding [20]. However, NE knockout mice are resistant to HFD-induced macrophage accumulation [18]. Together with current study, our data support the notion that NE-mediated vascular injury plays a significant role in initiating and propagating obesity-related inflammation, such as macrophage infiltration in adipose tissues. Thus, NE is a potential drug target for controlling vascular injury and subsequent inflammation in obesity. It is also worth noting that NE’s effect on hEC permeability is more potent than the synthetic PAR2 agonist in our experiments, suggesting that NE is a natural activator of PAR2 in vascular endothelial cells.

This study attempted to elucidate the molecular mechanism by which NE regulates vascular permeability. Multiple studies have suggested that NE activates TLR4 and PAR2 signaling leading inflammatory signaling and activation Erk1/2 MAP kinase [8,39,40,41,42,43,44,45]. Yet, inhibition of NFκB, TLR4, or MAP kinase signaling did not alter NE-induced F-actin formation and VE-cadherin loss in hECs. Remarkably, NE directly stimulates PAR2 endocytosis, whereas PAR2 inhibitor blocks NE’s effect. The effect of NE on PAR2 endocytosis was mimicked by a PAR2-activating peptide and another PAR2-activating protease trypsin, suggesting NE is a PAR2 activator in endothelial cells. Our finding is consistent with the report that NE cleaves PAR2 at the amino terminus and functions as a biased activator of PAR2 [41,43,62]. PAR2 endocytosis may be a common regulatory mechanism for the activated G-protein-coupled receptor as previously reported [44,63,64,65,66]. NE dramatically stimulates F-actin formation, whereas inhibitors of PAR2 and ROCK prevent NE-induced actin-rearrangement in hECs. NE also stimulates the phosphorylation of MLC and MYPT1 in a manner dependent on the activation of PAR2 and ROCK. MYPT1 is a critical phosphatase that dephosphorylates MLC. ROCK can phosphorylate and inhibit MYPT1 activity, leading to increased MLC phosphorylation and myosin activation [49]. PAR2 also activates MLCK, a kinase that phosphorylates MLC [67]. Inhibition of MLCK also partially blocked NE-induced F-actin formation and MLC phosphorylation. Our data suggest that NE regulates the actomyosin through activating the PAR2–ROCK and PAR2–MLCK pathways.

VE-cadherin is a critical component of intercellular junctions in vascular endothelial cells. It plays a vital role in regulating vascular permeability [23,24]. VE-cadherin is known to be regulated by proteasome activity [68,69]. The organization of the actomyosin cytoskeleton tightly controls VE-cadherin-mediated cell–cell adhesions. The cytoplasmic domain of VE-cadherin is anchored to the actin cytoskeleton via p120-, α-, and β-catenins at cell–cell junctions [26,27,28]. Our results revealed that NE increases F-actin formation and MLC phosphorylation and dramatically reduces VE-cadherin protein levels in hECs. NE-induced degradation of VE-cadherin is prohibited by inhibitors of PAR2, ROCK, MLCK, or proteasome. Interestingly, our data revealed that the higher levels of p-MLC and the lower levels of VE-cadherin are in hECs. We also observed that actin-interacting β-catenin and p120-catenin protein levels were decreased in NE-treated hECs but reversed by proteasome inhibitor MG132. Actin-binding catenin proteins interact with and stabilize VE-cadherin at the adhesin junction [26,27,28]. NE-stimulated actomyosin activation and catenin protein reduction may destabilize VE-cadherin at the intercellular junctions, leading to VE-cadherin internalization and subsequent degradation by the proteasome in hECs.

In addition to its effect on VE-cadherin protein degradation, our data also revealed that NE represses VE-cadherin gene expression in cultured hECs. NE’s inhibitory effect on VE-cadherin gene expression was mimicked by a PAR2 agonist but was completely reversed by a PAR2 inhibitor. However, inhibition of either ROCK1/2 or MLCK did not alter NE’s inhibitory effect on VE-cadherin gene expression. A previous study reported that activated PAR2 is internalized and associated with β-arrestin-1 during endocytosis [44,63,64,65,66]. The latter is known to regulate VE-cadherin gene expression in endothelial cells [50,51]. Further studies are needed to dissect the molecular mechanism by which NE activates ROCK-independent PAR2 downstream signaling involved in the repression of VE-cadherin gene expression in vascular endothelial cells.

In summary, our data suggest that NE regulates vascular endothelial cell permeability by activating two distinctive PAR2 downstream signaling pathways. The ROCK/MLCK-dependent pathway regulates actomyosin cytoskeletal activity and VE-cadherin degradation. Activating PAR2 downstream signaling independent of ROCK also suppresses VE-cadherin gene expression. Together, NE signaling in vascular endothelial cells increases para-endothelial permeability to immune cells, accelerating inflammatory tissue damage, as observed in obese animal models.

## Data Availability

The data presented in this study are available within the article and Supplementary Materials and upon request.

## Supplementary Materials

The following supporting information can be downloaded at https://www.mdpi.com/article/10.3390/cells11152288/s1, Figure S1: Neutrophil elastase (NE) and NE inhibitor regulated VE-cadherin protein and F-actin formation in dose dependent manners: Figure S2: Effects of various inhibitors on VE-cadherin expression and F-actin formation in vascular endothelial cells; Figure S3: NE and trypsin regulated VE-cadherin expression and F-actin formation through activating protease-activated receptor 2 (PAR2); Figure S4: Activation of PAR2 signaling regulated VE-cadherin, actomyosin cytoskeleton, and permeability in hECs; Figure S5: Flow cytometry gating strategies for identification of CD45^+^ immune cells, adipose tissue microphages (ATMs), and neutrophils in stromal vascular cells (SVCs).
