# Supplementary material for: Neutrophil Elastase Increases Vascular Permeability and Leukocyte Transmigration in Cultured Endothelial Cells and Obese Mice
Source: Cells. 2022 Jul 25;11(15):2288. doi: 10.3390/cells11152288 (PMC9332277; doi:10.3390/cells11152288)
Supplement: Supplementary file 1 [file cells-11-02288-s001.zip › cells-1720818-supplementary.pdf]

Supplemental Figures

Figure S1

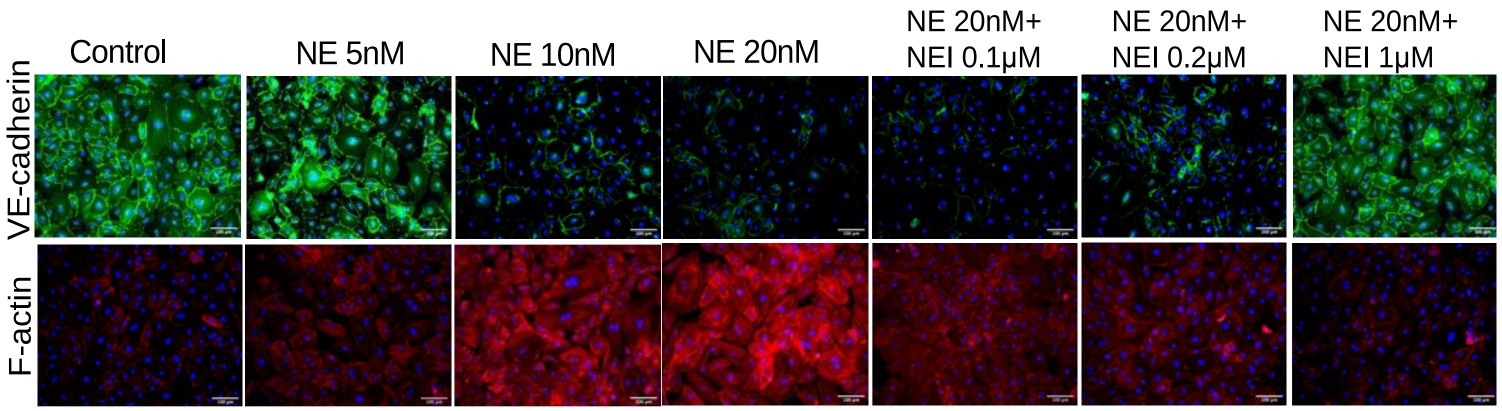

Figure S1. Neutrophil elastase (NE) and NE inhibitor regulated VE-cadherin adhesion protein and F-actin formation in dose dependent manners. Confluent hECs were treated with different concentrations of NE (5,10 & 20 nM) with or without NE inhibitor GW311616A (NEI, 0.1, 0.2, & 1 μM) for 16 hours. Cells were fixed and fluorescence stained with VE-cadherin antibody (Alexa-Fluor 488, green, top panel), and F-actin staining with phalloidin-iFluor 594 (Red, lower panel) to analyze the distribution of adhesion molecule VE-cadherin and F-actin. Images were taken at 20x magnification (Scale bar, 100 μm). Representative images are chosen from three independent experiments. NE: Neutrophil Elastase, NEI: Neutrophil Elastase inhibitor. *Also see main Figure 1.*

Figure S2

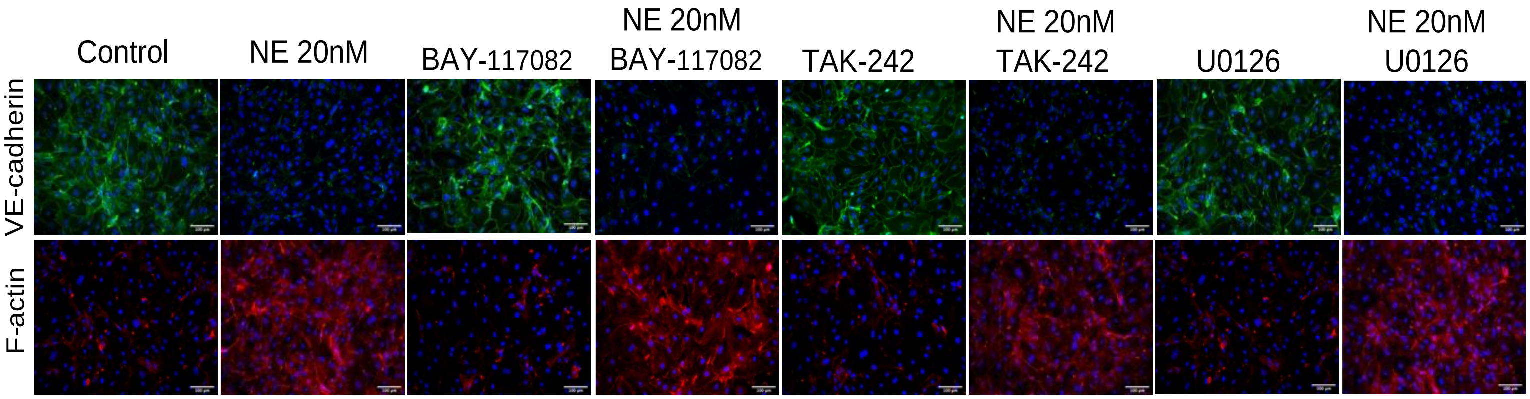

Figure S2. Effects of various inhibitors on VE-cadherin expression and F-actin formation in vascular endothelial cells. hECs were treated with inhibitors of NFκB (BAY-117082, 5 μM), TLR4 (TAK-242, 5 μM), and ERK1/2 (U0126, 5 μM) with or without NE (20 nM) for 16 hours. Cells were fixed and immunofluorescence staining of VE-cadherin (green, top panel, antibody from Cell signaling Inc) and F-actin with phalloidin-iFluor 594 (red, lower panel) were performed. Images were taken at 20x magnification (Scale bar, 100 μm). Representative images are chosen from three independent experiments. *Also see main Figure 2.*

Figure S3

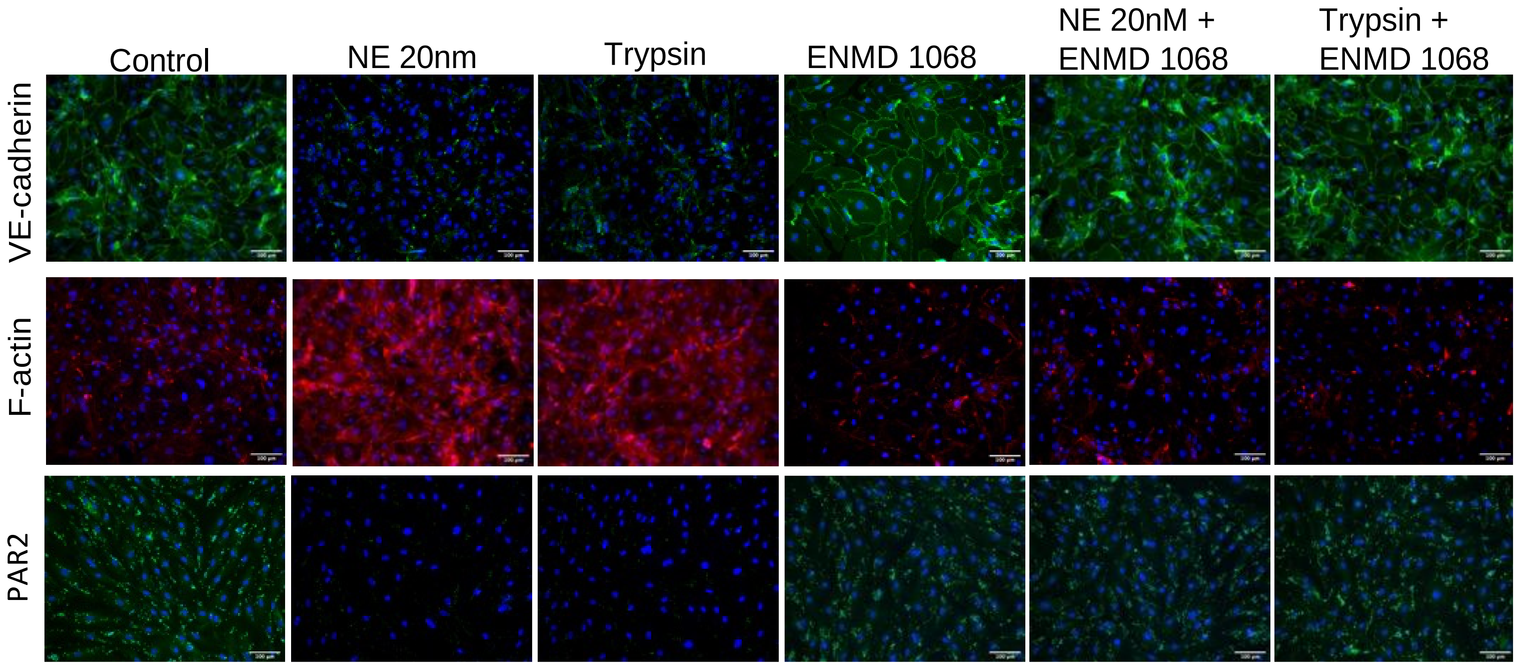

Figure S3. NE and trypsin regulated VE-cadherin expression and F-actin formation through activating protease-activated receptor 2 (PAR2). Immunofluorescence images of VE-cadherin (green, top panel), F-actin (Red, middle panel), PAR2 (green, lower panel), and nuclei counterstaining DAPI (blue). hEC cells were grown to confluence and treated with NE (20 nM) or Trypsin (100 nM, a known PAR2 agonist) in the presence or absence of PAR2 inhibitor (ENMD-1068, 250 µM) for 16 hours. Images were taken at 20x magnification using a fluorescence microscope, scale bar; 100 µm. NE: Neutrophil Elastase. VE-cadherin antibody was from Cell signaling Inc. *Also see main Figure 2.*

Figure S4

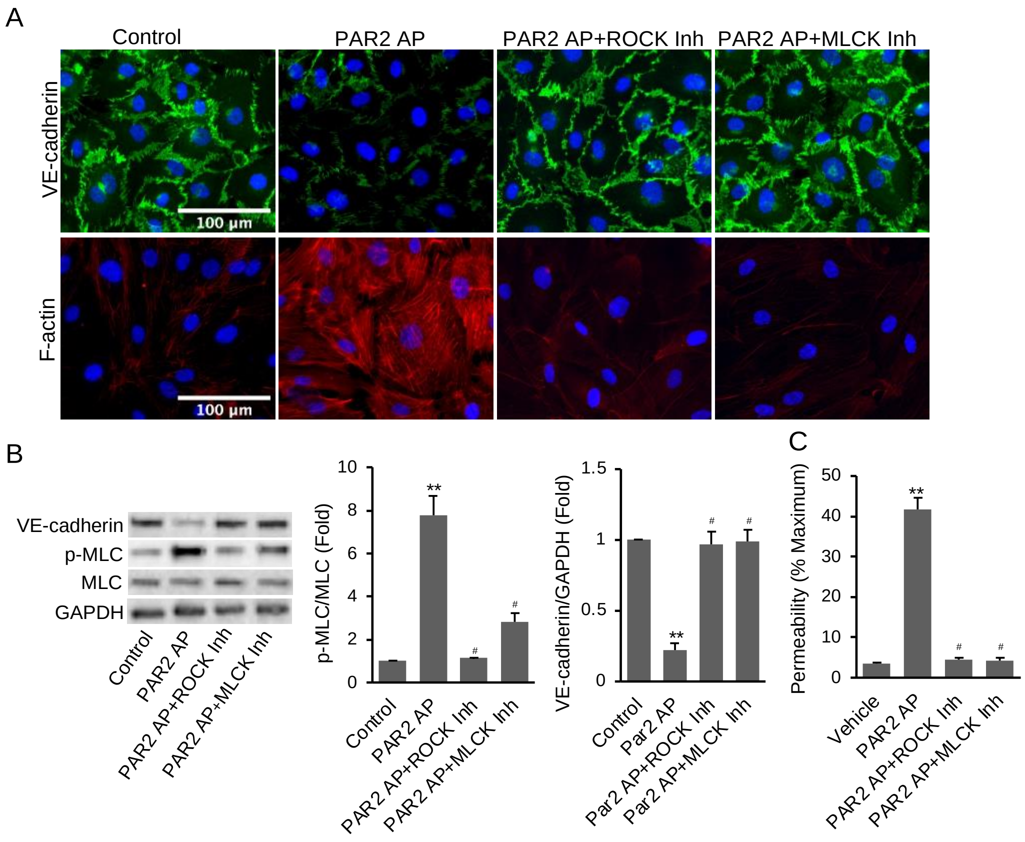

Figure S4. Activation of PAR2 signaling regulated VE-cadherin, actomyosin cytoskeleton, and permeability in hECs. (A) Fluorescence images of VE-cadherin (Green) and F-actin (Red). hECs were treated with PAR2 agonist (7.5 μM) alone and in combination with ROCK inhibitor (Y27632, 5 μM) and MLCK inhibitor (peptide 18, 5 μM) for 16 hours. The fluorescence images are representative of three independent experiments (Scale bar, 100 μm). **(B)** Western blot analysis of VE-cadherin and p-MLC (Ser19) in hECs treated with PAR2 AP and ROCK inhibitor or MLCK inhibitor. **(C)** Percentage of permeability in hEC monolayer treated with PAR2 agonist and different inhibitors. The values are expressed as mean±SD of three independent experiments. **$p < 0.01$ vs vehicle control group, and $^{\#}p < 0.05$ vs PAR2 AP group. PAR2 AP: PAR2 agonist (PAR2 (I-6) amide trifluro acetate salt) (7.5μM), ROCK Inh: ROCK inhibitor, MLCK inh: MLCK inhibitor. *Also see main Figures 2, 3, & 4.*

Figure S5

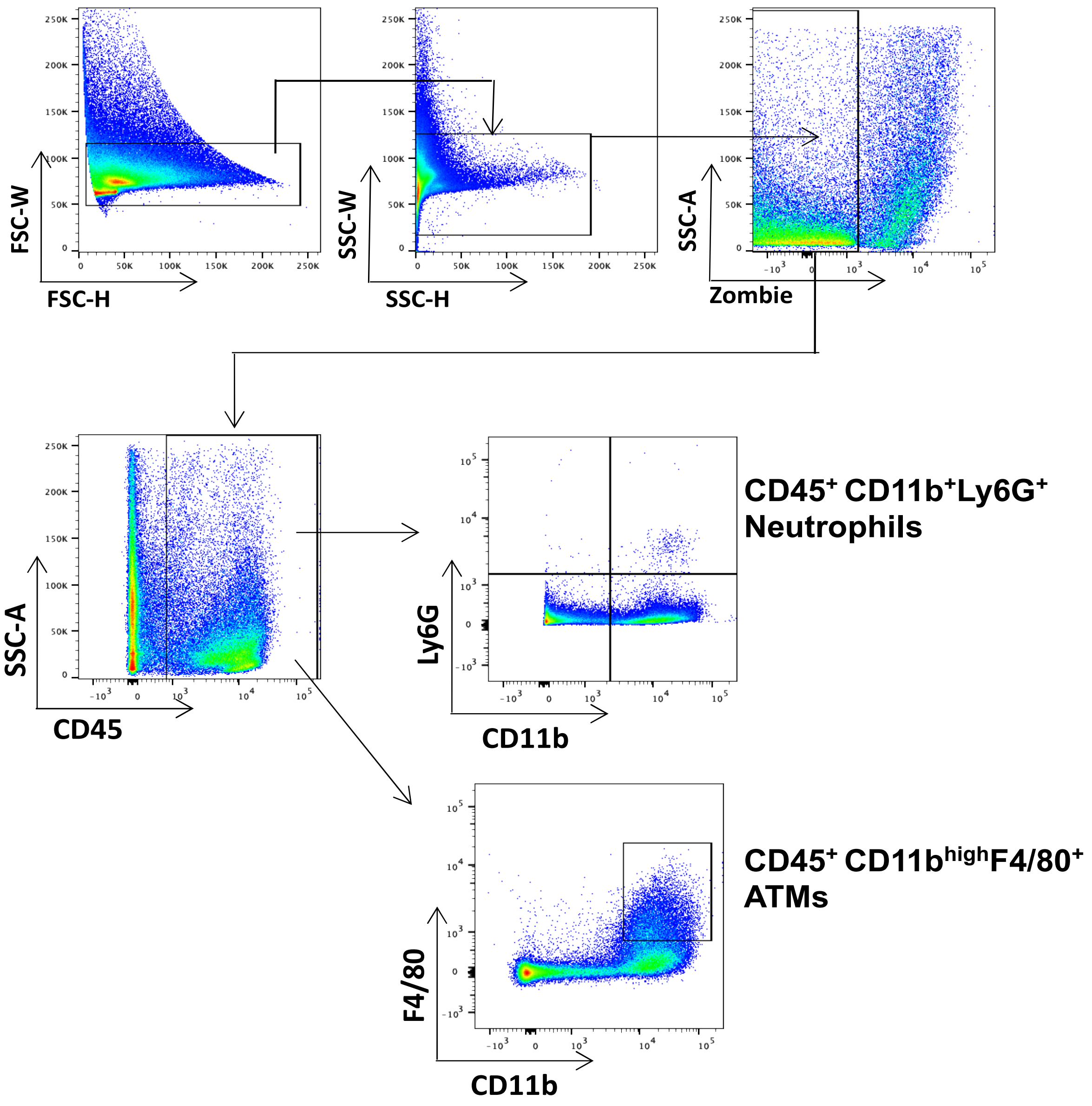

Figure S5. Flow cytometry gating strategies for identification of CD45+ immune cells, adipose tissue microphages (ATM), and neutrophils in stromal vascular cells (SVCs). Isolated SVF cells were first gated for singlet cells (FSC-H vs. FSC-W and SSC-H vs. SSC-W) and then analyzed for their uptake of Zombie-Aqua stain to determine live versus dead cells. In live cells, $CD45^{+}$ cells were identified and used for further analyses of adipose tissue macrophages (ATMs) and neutrophils. ATMs were identified as $CD45^{+}CD11b^{high}F4/80^{+}$ cells and neutrophils were identified as $CD45^{+}Ly6G^{+}CD11b^{+}$ cells. *Also see main Figures 7.*
